# Sweet-Potato-Vine-Based High-Performance Porous Carbon for Methylene Blue Adsorption

**DOI:** 10.3390/molecules28020819

**Published:** 2023-01-13

**Authors:** Wenlin Zhang, Yuhong Zhao, Qinhong Liao, Zhexin Li, Dengwei Jue, Jianmin Tang

**Affiliations:** 1 Chongqing Key Laboratory of Economic Plant Biotechnology, College of Landscape Architecture and Life Science/Institute of Special Plants, Chongqing University of Arts and Sciences, Yongchuan, Chongqing 402160, China; 2 College of Food Science, Southwest University, Beibei, Chongqing 400716, China; 3 College of Biology and Food Engineering, Chongqing Three Gorges University, Wanzhou, Chongqing 404199, China

**Keywords:** porous carbon, sweet potato vine, adsorption, methylene blue

## Abstract

In this study, sweet-potato-vine-based porous carbon (SPVPC) was prepared using zinc chloride as an activating and pore-forming agent. The optimised SPVPC exhibited abundant porous structures with a high specific surface area of 1397.8 m^2^ g^−1^. Moreover, SPVPC exhibited excellent adsorption characteristics for removing methylene blue (MB) from aqueous solutions. The maximum adsorption capacity for MB reached 653.6 mg g^−1^, and the reusability was satisfactory. The adsorption kinetics and isotherm were in good agreement with the pseudo-second-order kinetics and Langmuir models, respectively. The adsorption mechanism was summarised as the synergistic effects of the hierarchically porous structures in SPVPC and various interactions between SPVPC and MB. Considering its low cost and excellent adsorption performance, the prepared porous carbon is a promising adsorbent candidate for dye wastewater treatment.

## 1. Introduction

Dye wastewater has attracted wide attention, with approximately 100 tons of dye discharged into natural water bodies annually, causing serious water pollution and ecosystem destruction [1]. Methylene blue (MB) is a synthetic cationic dye and the most commonly used organic dye in artificial synthesis [2]. It poses serious threats to human health, such as cyanosis, heart disease, eye burns, tissue necrosis, nausea, diarrhoea, emesis, gastritis [3,4], and negatively affects the growth of aquatic plant and animals [5]. Moreover, MB is very stable and poorly degradable in wastewater under natural conditions [6]. Therefore, there is an urgent need to eliminate MB from wastewater using simple and efficient methods.

Currently available techniques, such as membrane separation, biodegradation, adsorption, electrochemical, photocatalysis, oxidation, and flocculation, have been employed to purify dye wastewater [7]. In contrast, adsorption technology is widely applied because of its high efficiency, low-cost, easy operation and the extensiveness of available materials [1]. Various adsorbents, such as carbon materials, metal nanomaterials, biomass, hydrogels, graphene, polymers, metal–organic frameworks, and their derivatives, have been explored for dye adsorption [8]. Among them, porous carbon (PC) is considered a potential dye adsorbent owing to its abundant pore structure, high specific surface area, and excellent adsorbability [9]. In recent years, the preparation of PC through renewable and cost-effective biomass as a basic material has arisen great interest, for example, rice husk [10], wheat straw [11], pomelo peel [12], cotton stalk [13], bagasse [14], etc. [15].

**Figure 1:**
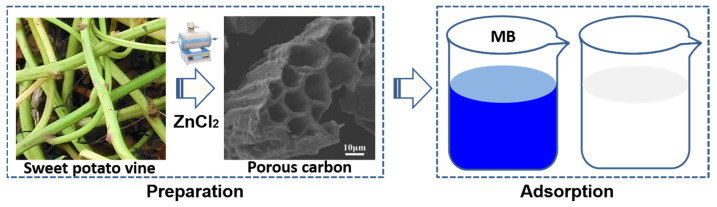
Schematic representation of the preparation and adsorption of SPVPC.

Sweet potato (*Ipomoea batatas* L.) is planted worldwide, and its tuberous root is usually used as a food source [16]. Sweet potato vine (SPV) is the aboveground biomass of sweet potatoes as a by-product of a farm. A large amount of sweet potato vine is produced annually in the field. A small part of them is used as animal feed, and most of them are discarded as agricultural waste and underutilised [17,18], which leads to resource waste and environmental pollution. Dried SPV is rich in cellulose (33.01%), hemicellulose (12.25%), and lignin (7.85%) [18]. Therefore, the use of SPV as a raw material to develop PC is worth considering. Gao et al. prepared SPV-based N- and S-doped carbons at 800 °C to catalyse the oxygen reduction reaction [19]. However, their preparation temperature was relatively high, and the porous morphology and specific surface area required further improvement. To the best of our knowledge, high-quality SPV-based PC (SPVPC) as an MB adsorbent has not yet been reported. In this study, as shown in Figure 1, SPVPC was developed using zinc chloride (ZnCl_2_) as an activating and pore-forming agent, and its structural characteristics and adsorption performance towards MB were analysed in detail.

## 2. Results and Discussion

### 2.1. Preparation Parameters of SPVPC

In this study, SPVPC was prepared using ZnCl_2_ as the pore-forming agent. ZnCl_2_ can catalyse the degradation, dehydration, and carbonisation of cellulose-rich materials, leading to the charring and aromatisation of the carbon skeleton and the formation of pore structures [20]. Therefore, the preparation impact factors of the mass ratio (MR) of ZnCl_2_ to SPV, as well as the carbonisation temperature (T) and time (t), were investigated using a single factor methodology to obtain high-performance SPVPC. As seen in Figure S1A, *q*_e_ increased sharply with the increasing MR until 2:1, with the highest value being 299.6 mg g^−1^, but it slightly decreased when the MR was over 2:1. Moreover, the effect of T on *q*_e_ showed a similar pattern and *q*_e_ reached a maximum value of 299.2 mg g^−1^ at 500 °C (Figure S1B). A higher temperature of over 500 °C reduced *q*_e_ because it could damage the pore structure of carbon, reducing the specific surface area [21]. Additionally, when t was prolonged to 1 h, *q*_e_ reached its highest value of 299.7 mg g^−1^ and then decreased (Figure S1C). Therefore, MR = 2:1, T = 500 °C, and t = 1 h were selected as the optimal preparation conditions for SPVPC.

### 2.2. Physicochemical Properties of SPVPC

**Figure 2:**
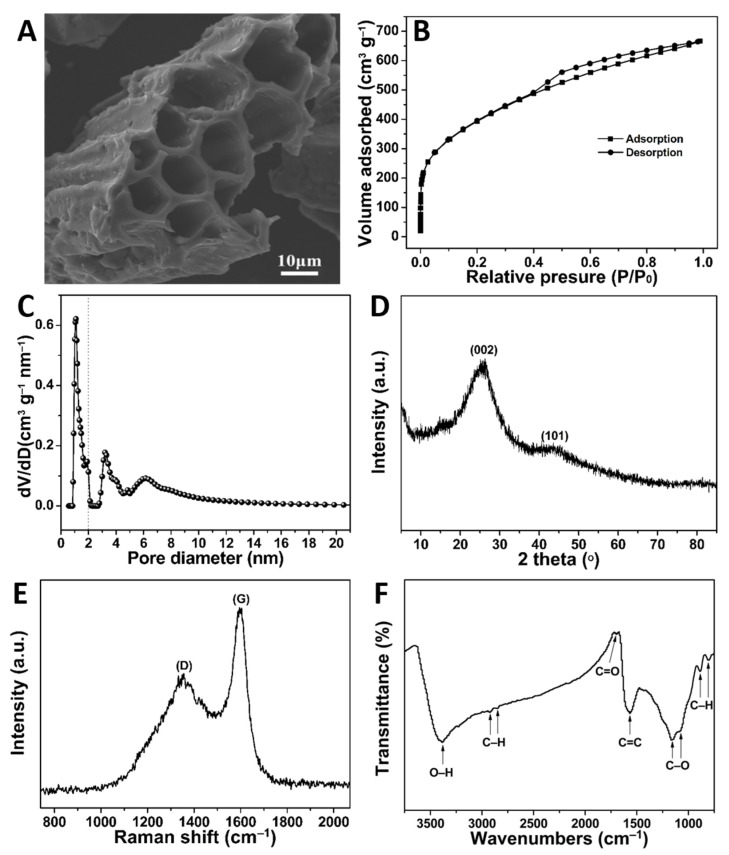
(**A**) SEM image, (**B**) nitrogen adsorption–desorption isotherm, (**C**) DFT pore size distribution curve, (**D**) XRD pattern, (**E**) Raman spectrum, and (**F**) FTIR spectrum of SPVPC.

The surface morphology of the optimised SPVPC was observed using SEM. As shown in Figure 2A, SPVPC exhibited a porous grid-pipe-like shape with abundant macropore structures. Furthermore, the mesopore and micropore properties, pore size distribution, and specific surface area were studied using the Brunauer–Emmett–Teller (BET) and density function theory (DFT) methods. Figure 2B showed that the nitrogen adsorption–desorption isotherm of SPVPC exhibited a typical IV adsorption isotherm according to the IUPAC classification, indicating the existence of micropores and mesopores in SPVPC [4]. An adsorption hysteresis loop in the curve at 0.4 < P/P_0_ < 1.0 demonstrated that SPVPC had abundant mesopores [12]. Meanwhile, the curve increased sharply at P/P_0_ < 0.01, suggesting the presence of rich micropores in the SPVPC [1]. The DFT pore size distribution also revealed that SPVPC had generous micropores and mesopores with the average pore diameter of 2.9 nm (Figure 2C). Combining the SEM results, SPVPC possessed a large number of pore structures with a total pore volume of 1.03 cm^3^ g^−1^. Notably, the pore structures could accelerate the diffusion of MB molecules on the surface of SPVPC rapidly and entrap more MB molecules [22]. Surprisingly, the specific surface area of SPVPC reached 1397.8 m^2^ g^−1^ owing to its rich porous structure and was higher than those of other recently reported PCs [12,13,23,24,25], especially the reported SPV-derived carbon (884.9 m^2^ g^−1^) [19].

The crystallographic characteristics of the SPVPC were explored using the XRD pattern shown in Figure 2D. The sample exhibited two broad diffraction peaks at around 26° and 44°, consistent with the (002) and (100)/(101) crystal planes of typical graphitic carbon (JCPDS card no. 75-1621) [26]. The (002) diffraction revealed that the carbons were randomly oriented carbon layers with many defects, while the (100)/(101) diffraction indicated a turbostratic structure with low crystallinity [26,27]. These turbostratic structures favoured the adsorption of dye molecules [28]. To further investigate the structure of the SPVPC, its Raman spectrum was recorded (Figure 2E). The peak at 1356 cm^−1^ (D-band) is generally attributed to disordered and defective carbons with the breathing mode of the A1g symmetry, and 1595 cm^−1^ (G-band) corresponds to ordered graphitic structures with the zone centre of the E2g mode [19,26]. Furthermore, the calculated intensity ratio of the D and G bands (ID/IG) was 0.7, which was less than that of pristine graphene (approximately 1.4), indicating that SPVPC had abundant amorphous carbon structures [12,19]. Additionally, the full width at half-maximum of the D band was much larger than that of the G band, demonstrating many defects in SPVPC [26], which was associated with the XRD analysis. The surface functional groups of the SPVPC were analysed using FTIR spectroscopy. Figure 2F shows the characteristic bands at 3385 cm^−1^, 2926 cm^−1^ and 2860 cm^−1^, 1697 cm^−1^, 1568 cm^−1^, 1165 cm^−1^ and 1070 cm^−1^, 889 cm^−1^ and 812 cm^−1^, ascribed to the stretching vibrations of O–H (hydroxyl), C–H (methyl and methylene), C=O (carbonyl and carboxylic), C=C (benzene ring), C–O (ester, ether and phenol), and C–H (benzene ring), respectively [1,29,30,31]. Therefore, it can be concluded that SPVPC possessed abundant functional groups, ascribed to the carbonisation of SPV during the heat-treatment process at a relatively low temperature of 500 °C [12,32]. However, the higher temperature (600–700 °C) could destroy most functional groups of porous carbon according to our previous report [32]. Importantly, these groups played key roles in dye adsorption via various interactions [33].

### 2.3. Adsorption and Reusability Properties of SPVPC

**Figure 3:**
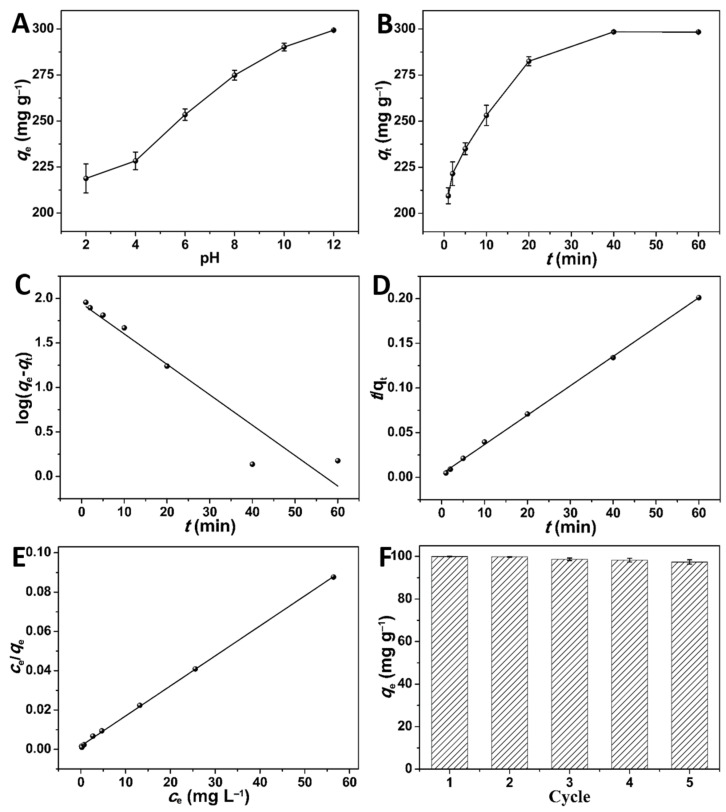
Effect of (**A**) pH (*c*_0_: 300 mg L^−1^, 120 min, 25 °C), (**B**) adsorption time (*c*_0_: 300 mg L^−1^, pH: 12, 25 °C), (**C**) PFO, and (**D**) PSO kinetics; (**E**) Langmuir isotherm and (**F**) reusability of MB adsorption on SPVPC.

Based on previous reports on MB adsorption, pH was considered a critical factor influencing the adsorption process by altering the chemical structure and surface of MB and the adsorbent [34]. As shown in Figure 3A, with the solution pH increasing from 2 to 12, *q*_e_ gradually increased from 218.8 mg g^−1^ to 299.4 mg g^−1^. MB is a cationic dye with a positive charge in an aqueous solution [12], and the SPVPC surface is negative at a pH > 4.1 (pH_zero charge point_, Figure S2A) [35,36]. As the pH increased from 4.1 to 12, *q*_e_ increased sharply owing to the reinforced electrostatic interaction between SPVPC and MB. When pH < 4.1, electrostatic repulsion between the positively charged SPVPC surface and MB occurred [35,36], and excess H^+^ competed with MB for the surface adsorption sites of SPVPC, leading to a lower *q*_e_ [2]. However, *q*_e_ could reach 218.8 mg g^−1^ at pH 2, indicating that adsorption was regulated by electrostatic interaction and other interactions, such as π–π attraction, and hydrogen bonding between SPVPC and MB molecules. Moreover, *q*_e_ increased with increasing *c*_0_ (Figure S2B), because a high *c*_0_ could improve the contact area between the MB molecules and SPVPC and provide a large driving force to accelerate MB mass transfer [2]. Additionally, a high adsorption temperature promoted *q*_e_ to a certain extent, involving an endothermic process (Figure S2C). Surprisingly, the MB adsorption on SPVPC was quick (209.5 mg g^−1^ min^−1^) within 1 min (Figure 3B), ascribed to the numerous unoccupied adsorption sites and the decrease of MB diffusion resistance by pores. Subsequently, the adsorption rate decreased gradually owing to the occupancy of adsorption sites on the SPVPC surface by MB molecules, which slowed the internal diffusion [26]. After 40 min, the adsorption reached an equilibrium with *q*_e_ of 298.5 mg g^−1^, revealing that SPVPC possessed a high adsorption efficiency for MB.

The PFO and PSO kinetics were analysed to investigate the adsorption process of MB by SPVPC (Figure 3C,D; Table S1). Compared with PFO, PSO had a higher correlation coefficient (*R*^2^ = 0.9993), and its calculated *q*_e,cal_ was consistent with the experimental *q*_e,exp_, revealing that PSO was more suitable for explaining the adsorption process, and chemisorption mainly dominated the adsorption behaviour of SPVPC towards MB [1]. Furthermore, the Langmuir and Freundlich isotherms were employed to determine the adsorption characteristics at equilibrium (Figure 3E and Figure S2D; Table S2). It was found that the *R*^2^ (0.9995) of Langmuir was much higher than that of Freundlich (0.8423), suggesting that the adsorption process fit the Langmuir isotherm and a monolayer coverage of MB molecules occurred on the SPVPC surface, which was consistent with previously reported PCs [1,12,32]. From the Langmuir isotherm, the calculated *q*_m_ of 653.6 mg g^−1^ was higher than that of most reported adsorbents (Table S3). Additionally, the reusability of SPVPC is an important factor in assessing their practical application. As shown in Figure 3F, the regenerated SPVPC maintained a high *q*_e_ of 97.3 mg g^−1^ after five cycles, and the *q*_e_ decreased by only 1.5%, indicating that SPVPC had excellent reusability and stability for MB removal. Therefore, the prepared SPVPC can be used as a potential regenerable adsorbent for dye wastewater cleaning.

### 2.4. Adsorption Mechanism of SPVPC towards MB

**Figure 4:**
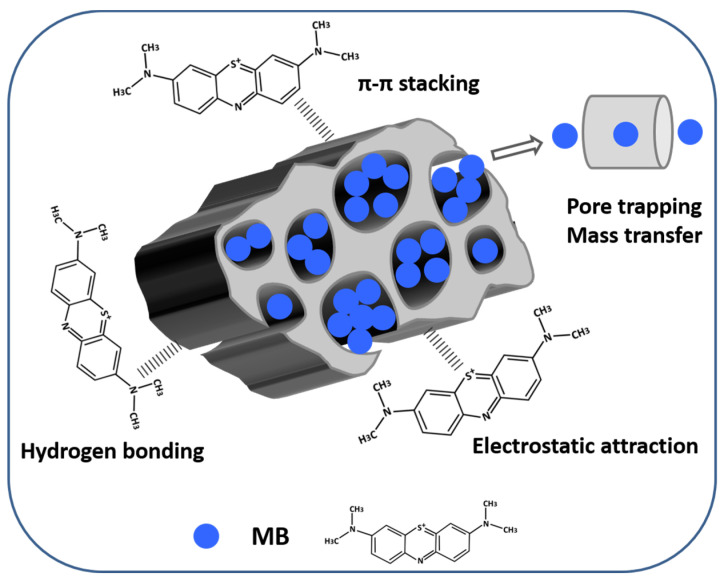
Adsorption mechanism diagram of SPVPC towards MB.

The MB adsorption mechanism of the SPVPC is summarised in Figure 4. According to the above PSO kinetics and Langmuir isotherm results, the adsorption process of SPVPC towards MB was mainly controlled by chemical forces and a monolayer adsorption [1]. Importantly, the rich hierarchical porous structures of SPVPC provided sufficient active adsorption sites to trap more MB molecules and offered available channels to decrease the migration resistance and improve the mass transfer process [12,22,30]. The adsorption was driven by the strong electrostatic attraction between the negatively charged functional groups on the SPVPC surface and the positively charged MB molecules [1,33]. Furthermore, hydrogen bonding interaction was a possible factor for adsorption, which occurred between the H atoms on the SPVPC surface and the N atoms on MB molecules [33]. In addition, the π–π interaction formed by the aromatic turbostratic structures of SPVPC and the benzene rings of MB molecules was favourable for the adsorption process [1,12,32]. Therefore, the adsorption mechanism of SPVPC was clarified as the various interactions between SPVPC and the MB dye and the rich pores in SPVPC.

## 3. Materials and Methods

### 3.1. Materials

Sweet potato vines were collected from a sweet potato field in Yongchuan (Chongqing, China), dried, milled, and passed through a 60 mesh sieve to obtain a powder with a particle size ≤0.25 mm for the carbonisation experiments. MB was purchased from the Shanghai Aladdin Chemical Company (Shanghai, China). ZnCl_2_, hydrochloric acid (HCl), sodium hydroxide (NaOH), and other analytical reagents were purchased from the Chengdu Kelong Chemical Company (Sichuan, China).

### 3.2. Preparation of SPVPC

SPVPC was prepared using the ZnCl_2_ activation method. Typically, 1.0 g of SPV powder and 0.5–4 g of ZnCl_2_ were dispersed in 50 mL of water with vigorous magnetic stirring for 1 h. The mixtures were then dried using a vacuum freeze dryer. The dried samples were heated and carbonised in a tube furnace at 300–700 °C for 0.5–2.5 h at a heating rate of 5 °C min^−1^ under a nitrogen flow. After natural cooling, the obtained samples were ground and washed with a HCl solution (1 mol L^−1^) and ultrapure water to remove inorganic impurities [19,20]. Finally, the samples were dried at 50 °C in a vacuum drying oven.

### 3.3. Characterisations of SPVPC

The morphology was observed using a JSM-7800F scanning electron microscope (SEM, JEOL, Tokyo, Japan) at 10 kV. The specific surface area and pore characteristics were determined using a porosity analyser (Quantachrome, Boynton Beach, FL, USA). The crystalline structure was determined using an Ultima IV X-ray diffractometer (XRD, Rigaku, Tokyo, Japan) with Cu-Kα radiation (λ = 0.15 nm) and a scan speed of 4° min^−1^. The microcrystalline structure of graphite was characterised using a Renishaw inVia plus micro-Raman spectroscopy system (Renishaw, Wotton-under-Edge, UK). The surface groups were recorded on a Nicolet 6700 Fourier transform infrared spectrophotometer (FTIR, Thermo Fisher, Waltham, MA, USA).

### 3.4. Adsorption of SPVPC

Batch adsorption experiments were performed by adding 10 mg SPVPC into 10 mL of MB aqueous solutions with different initial concentrations (100–700 mg L^−1^), pH values (2–12), times (1–120 min), and temperatures (25–50 °C) in an incubation shaker at a speed of 200 rpm. The mixtures were then centrifuged, and their supernatant absorbances were measured using a TU-1901 UV–vis spectrophotometer (Persee, Beijing, China) at 664 nm. The final concentrations of MB were determined using the standard curve of absorbance and concentration. The formulas of the adsorption capacity, pseudo-first-order (PFO) and pseudo-second-order (PSO) kinetics, and Langmuir and Freundlich isotherms are as follows.

(1) $$q_{e}=\frac{\left(c_{0}-c_{e}\right)V}{m}$$

(2) $$\log\left(q_{e}-q_{t}\right)\;{=\log q}_{e}-\frac{k_{1}t}{2.303}$$

(3) $$\frac{t}{q_{t}}=\frac{1}{k_{2}q_{e}^{2}}+\frac{t}{q_{e}}$$

(4) $$\frac{c_{e}}{q_{e}}=\frac{c_{e}}{q_{m}}+\frac{1}{{bq}_{m}}$$

(5) $${\log q}_{e}=\log k+\frac{1}{n}{\log c}_{e}$$

where *q*_e_ (mg g^−1^) and *q*_t_ (mg g^−1^) are the adsorption capacities of SPVPC toward MB at equilibrium and any time t (min), respectively; *c*_0_ (mg L^−1^) and *c*_e_ (mg L^−1^) are the initial and equilibrium concentrations of MB, respectively; *V* (L) is the MB solution volume; *m* (g) is the SPVPC mass; *k*_1_ (min^−1^) and *k*_2_ (g mg^−1^ min^−1^) are the rate constants of the PFO and PSO kinetics, respectively; *q*_m_ (mg g^−1^) is the max adsorption capacity; *b* (L mg^−1^) and *k* ((mg g^−1^(L mg^−1^)^1/n^) are the constants of the Langmuir and Freundlich isotherms, respectively; and 1/*n* is the heterogeneity factor of the Freundlich isotherm.

### 3.5. Reusability of SPVPC

Typically, 10 mg of SPVPC was mixed with 10 mL of a 100 mg L^−1^ MB solution at 200 rpm for 60 min (pH = 12, 25 °C). After adsorption, the MB-adsorbed SPVPC was separated by centrifugation, and 10 mL of ethanol (pH = 2) was added for desorption through ultrasound treatment for 5 min. Finally, the desorbed SPVPC was isolated by centrifugation for the next cycle. This process was repeated five times.

## 4. Conclusions

In this study, SPVPC was successfully prepared using a facile ZnCl_2_ activation strategy. The obtained SPVPC possessed a high specific surface area, rich pores, strong adsorption ability, and excellent reusability for MB removal. The adsorption process fit pseudo-second-order kinetics and Langmuir isotherm models. The adsorption mechanism was considered to be the integrated effect of hierarchical pores in SPVPC and the various interactions between SPVPC and MB molecules. This study provides a novel and promising absorbent material for treating dye wastewater. Prospectively, the microorganism and enzyme are possible alternatives and ecofriendly porogens to treat the biomass precursor for preparing PC.

## Data Availability

Not applicable.

## Supplementary Materials

The following are available online at https://www.mdpi.com/article/10.3390/molecules28020819/s1. Figure S1: Effect of the preparation parameters of (A) MR (T: 500 °C, t: 1 h), (B) T (MR: 2:1, t: 1 h), and (C) t (MR: 2:1, T: 500 °C) of SPVPC towards MB adsorption (c0: 300 g L−1, pH = 12, 25 °C, 2 h), Figure S2: (A) Zero-charge point of SPVPC. Effect of (B) c0, (C) T, and (D) Freundlich isotherm for MB adsorption on SPVPC, Table S1: PFO and PSO kinetics parameters, Table S2: Langmuir and Freundlich isotherm parameters, Table S3: Comparison of the max adsorption capacities of MB on various adsorbents. References from [37,38,39,40,41,42,43,44,45] are from Supplementary Materials.
