# Supplementary material for: Sweet-Potato-Vine-Based High-Performance Porous Carbon for Methylene Blue Adsorption
Source: Molecules. 2023 Jan 13;28(2):819. doi: 10.3390/molecules28020819 (PMC9867065; doi:10.3390/molecules28020819)
Supplement: Supplementary file 1 [file molecules-28-00819-s001.zip › molecules-2144417-supplementary.pdf]

Supplementary Materials

Sweet-Potato-Vine-Based High-Performance Porous Carbon for Methylene Blue Adsorption

Wenlin Zhang [1,2], Yuhong Zhao [1,3], Qinhong Liao [1,*], Zhexin Li [1], Dengwei Jue [1] and Jianmin Tang [1]

[1] Chongqing Key Laboratory of Economic Plant Biotechnology, College of Landscape Architecture and Life Science/Institute of Special Plants, Chongqing University of Arts and Sciences, Yongchuan, Chongqing 402160, China
[2] College of Food Science, Southwest University, Beibei, Chongqing 400716, China
[3] College of Biology and Food Engineering, Chongqing Three Gorges University, Wanzhou, Chongqing 404199, China

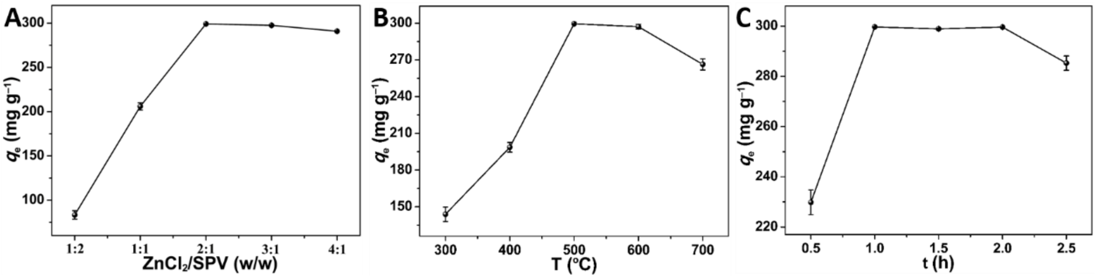

Figure S1. Effect of the preparation parameters of (**A**) MR (T: 500 °C, t: 1 h), (**B**) T (MR: 1:2, t: 1 h), and (**C**) t (MR: 1:2, T: 500 °C) of SPVPC towards MB adsorption (c_0: 300 g L^{-1}, pH=12, 25 °C, 2 h).

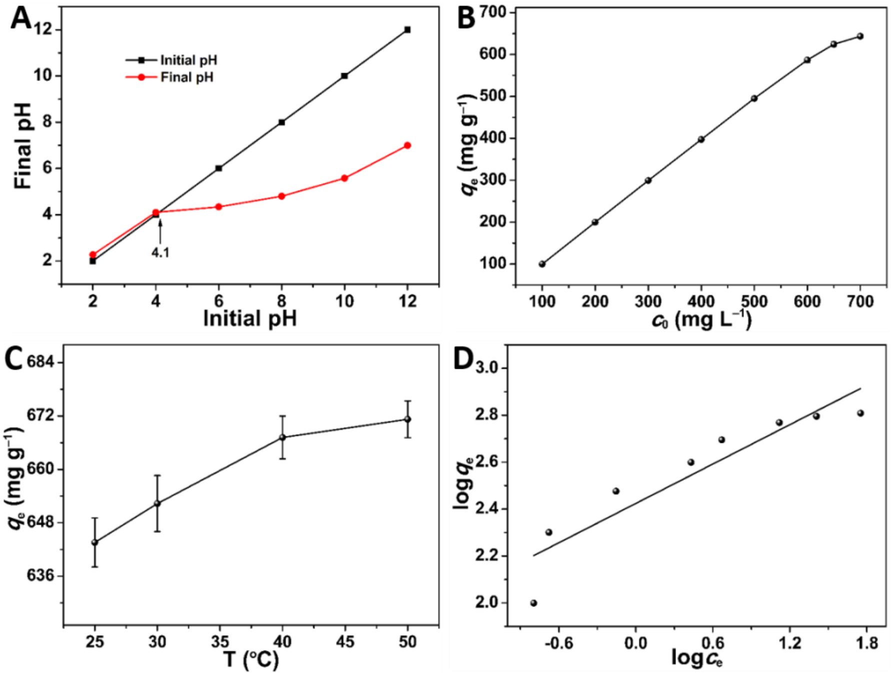

Figure S2. (A) Zero charge point of SPVPC. Effect of (B) c_0, (C) T, and (D) Freundlich isotherm for MB adsorption on SPVPC.

Table S1. PFO and PSO kinetics parameters.

Pseudo-first-order kinetic					Pseudo-second-order kinetic		
c_0 (mg L^{-1})	$q_{e,exp}$ (mg g^{-1})	$q_{e,cal}$ (mg g^{-1})	k_1 (min^{-1})	R^2	$q_{e,cal}$ (mg g^{-1})	k_2 (g mg^{-1} min^{-1})	R^2
300	299.8	87.6	0.08	0.9106	304.0	0.0028	0.9993

Table S2. Langmuir and Freundlich isotherm parameters.

Temperature (°C)	Langmuir			Freundlich		
	q_m (mg g^{-1})	b (L mg^{-1})	R^2	k	$1/n$	R^2
25	653.6	0.87	0.9995	265.66	0.28	0.8423

Table S3. Comparison of the max adsorption capacities of MB on various adsorbents.

Adsorbents	q_m (mg g^{-1})	References
Biochar microparticles	8.9	[37]
Polycatechol modified Fe_3O_4 magnetic nanoparticles	60.06	[38]
EDTAD-modified sugarcane bagasse	115.3	[39]
Ultrathin 2D MoS_2 nanosheets	146.43	[40]
Natural clay	202.13	[41]
Ginger straw derived porous carbons	345.0	[42]
Graphite oxide	351	[43]
Nanosheet MFI zeolite	476.19	[44]
Corncob-derived activated carbon	523.18	[45]
Cotton charcoal/chitosan biomass-based hydrogel	590.72	[46]
**Sweet potato vine derived porous carbon**	**653.6**	**This work**

References

[37] Lonappan, L., Rouissi, T., Das, R. K., Brar, S. K., Ramirez, A. A., Verma, M., ... & Valero, J. R. (2016). Adsorption of methylene blue on biochar microparticles derived from different waste materials. Waste Management, 49, 537-544.

[38] Hua, Y., Xiao, J., Zhang, Q., Cui, C., & Wang, C. (2018). Facile synthesis of surface-functionalized magnetic nanocomposites for effectively selective adsorption of cationic dyes. Nanoscale research letters, 13(1), 1-9.

[39] Xing, Y., Liu, D., & Zhang, L. P. (2010). Enhanced adsorption of Methylene Blue by EDTAD-modified sugarcane bagasse and photocatalytic regeneration of the adsorbent. Desalination, 259(1-3), 187-191.

[40] Qiao, X. Q., Hu, F. C., Tian, F. Y., Hou, D. F., & Li, D. S. (2016). Equilibrium and kinetic studies on MB adsorption by ultrathin 2D MoS_2 nanosheets. Rsc Advances, 6(14), 11631-11636.

[41] Bentahar, S., Dbik, A., El Khomri, M., El Messaoudi, N., & Lacherai, A. (2017). Adsorption of methylene blue, crystal violet and congo red from binary and ternary systems with natural clay: kinetic, isotherm, and thermodynamic. Journal of environmental chemical engineering, 5(6), 5921-5932.

[42] Zhang, W., Li, H., Tang, J., Lu, H., & Liu, Y. (2019). Ginger straw waste-derived porous carbons as effective adsorbents toward methylene blue. Molecules, 24(3), 469.

[43] Bradder, P., Ling, S. K., Wang, S., & Liu, S. (2011). Dye adsorption on layered graphite oxide. Journal of Chemical & Engineering Data, 56(1), 138-141.

[44] Ji, Y., Xu, F., Wei, W., Gao, H., Zhang, K., Zhang, G., ... & Zhang, P. (2021). Efficient and fast adsorption of methylene blue dye onto a nanosheet MFI zeolite. Journal of Solid State Chemistry, 295, 121917.

[45] Sun, Z., Qu, K., Cheng, Y., You, Y., Huang, Z., Umar, A., ... & Guo, Z. (2021). Corncob-derived activated carbon for efficient adsorption dye in sewage. ES Food & Agroforestry, 4, 61-73.

[46] Fan, X., Wang, X., Cai, Y., Xie, H., Han, S., & Hao, C. (2022). Functionalized cotton charcoal/chitosan biomass-based hydrogel for capturing Pb^{2+}, Cu^{2+} and MB. Journal of Hazardous Materials, 423, 127191.
